# Supplementary figures and images for: Ferric reductase-related proteins mediate fungal heme acquisition
Source: eLife. 2022 Oct 6;11:e80604. doi: 10.7554/eLife.80604 (PMC9635878; doi:10.7554/eLife.80604)

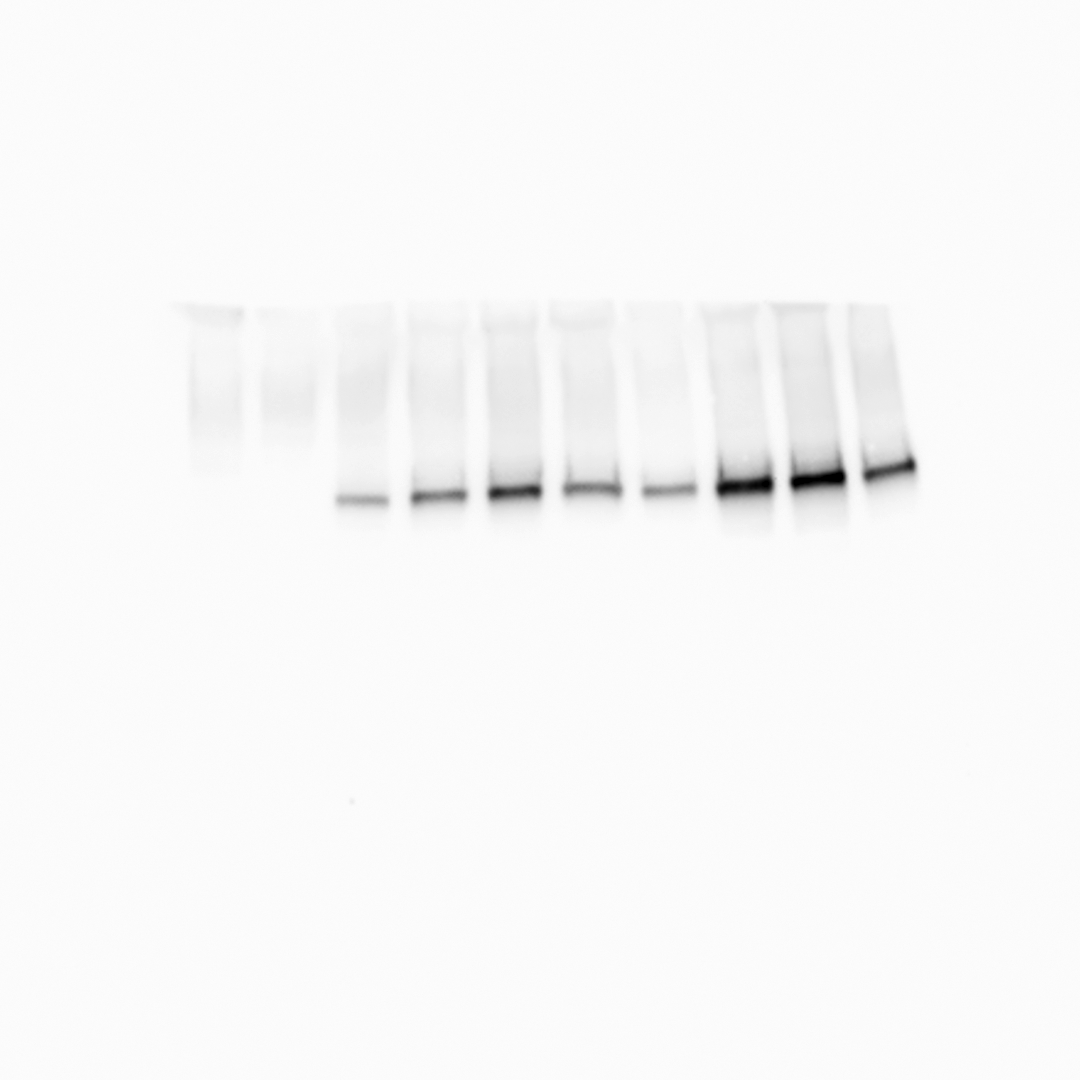

Supplement: Figure 4—figure supplement 2—source data 1. [file elife-80604-fig4-figsupp2-data1.zip › Fig 4 sup 2 /Fig 4 sup 2 A lower panel raw.Tif]

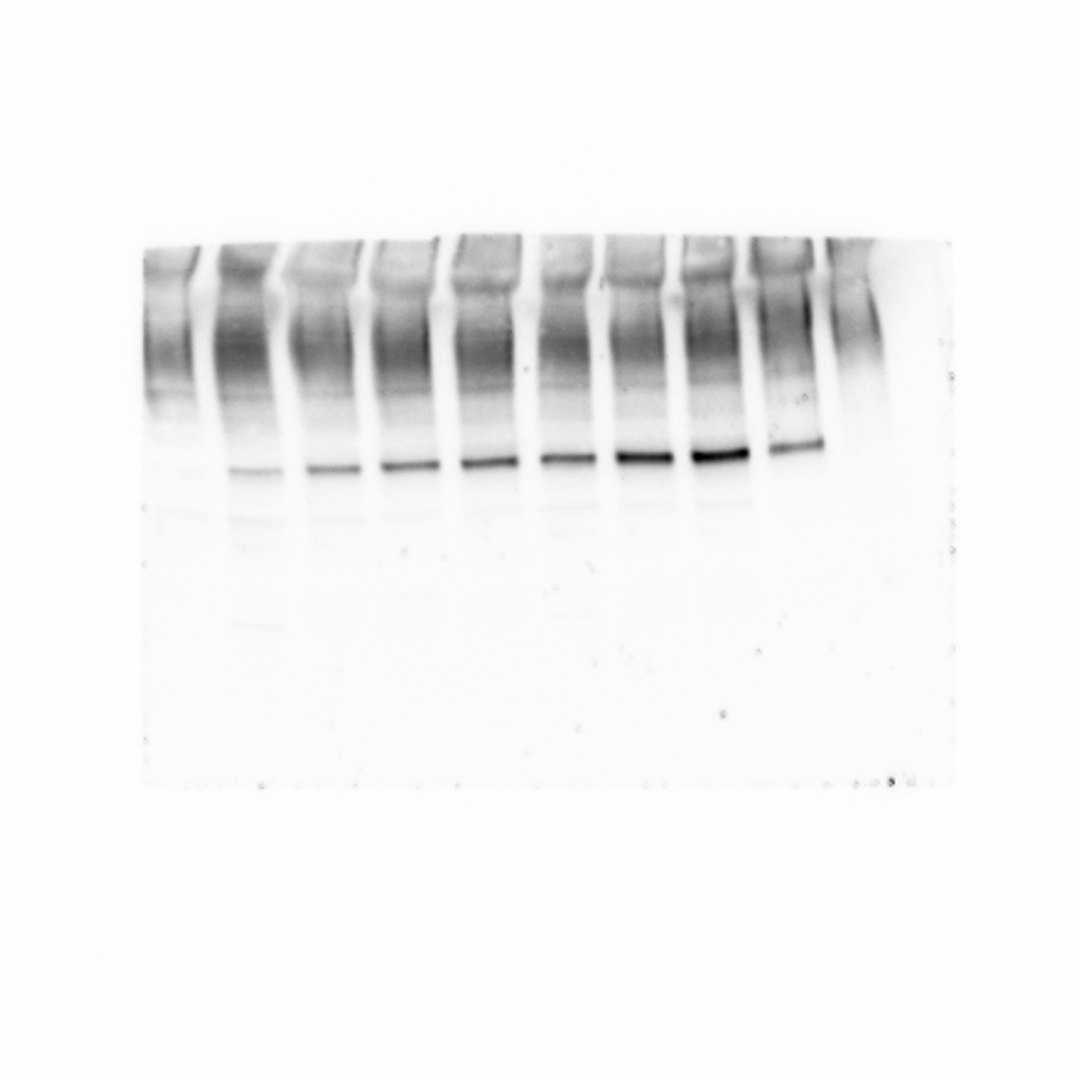

Supplement: Figure 4—figure supplement 2—source data 1. [file elife-80604-fig4-figsupp2-data1.zip › Fig 4 sup 2 /Fig 4 sup 2 A top panel raw.Tif]

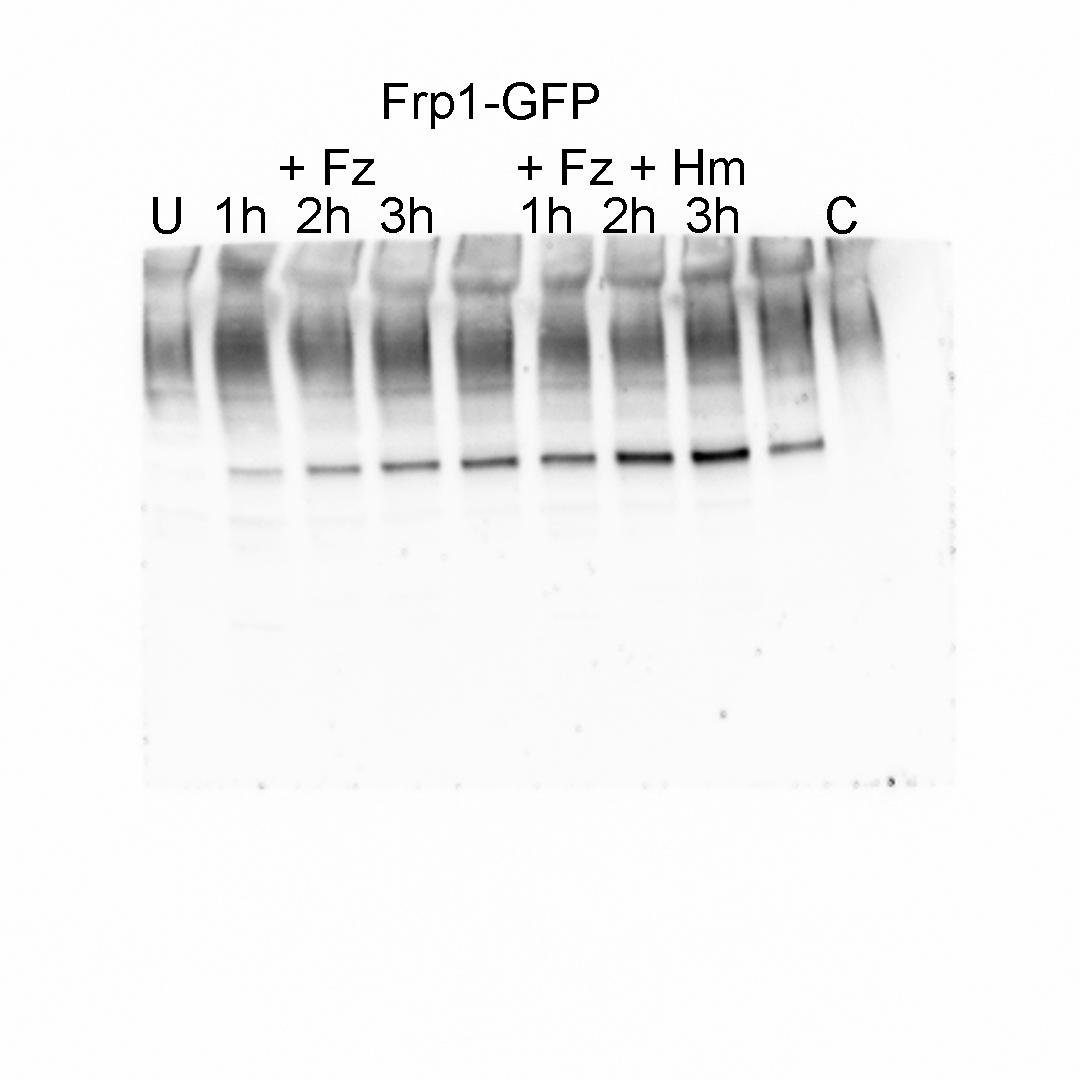

Supplement: Figure 4—figure supplement 2—source data 1. [file elife-80604-fig4-figsupp2-data1.zip › Fig 4 sup 2 /Fig 4 sup 2 A top panel marked.tif]

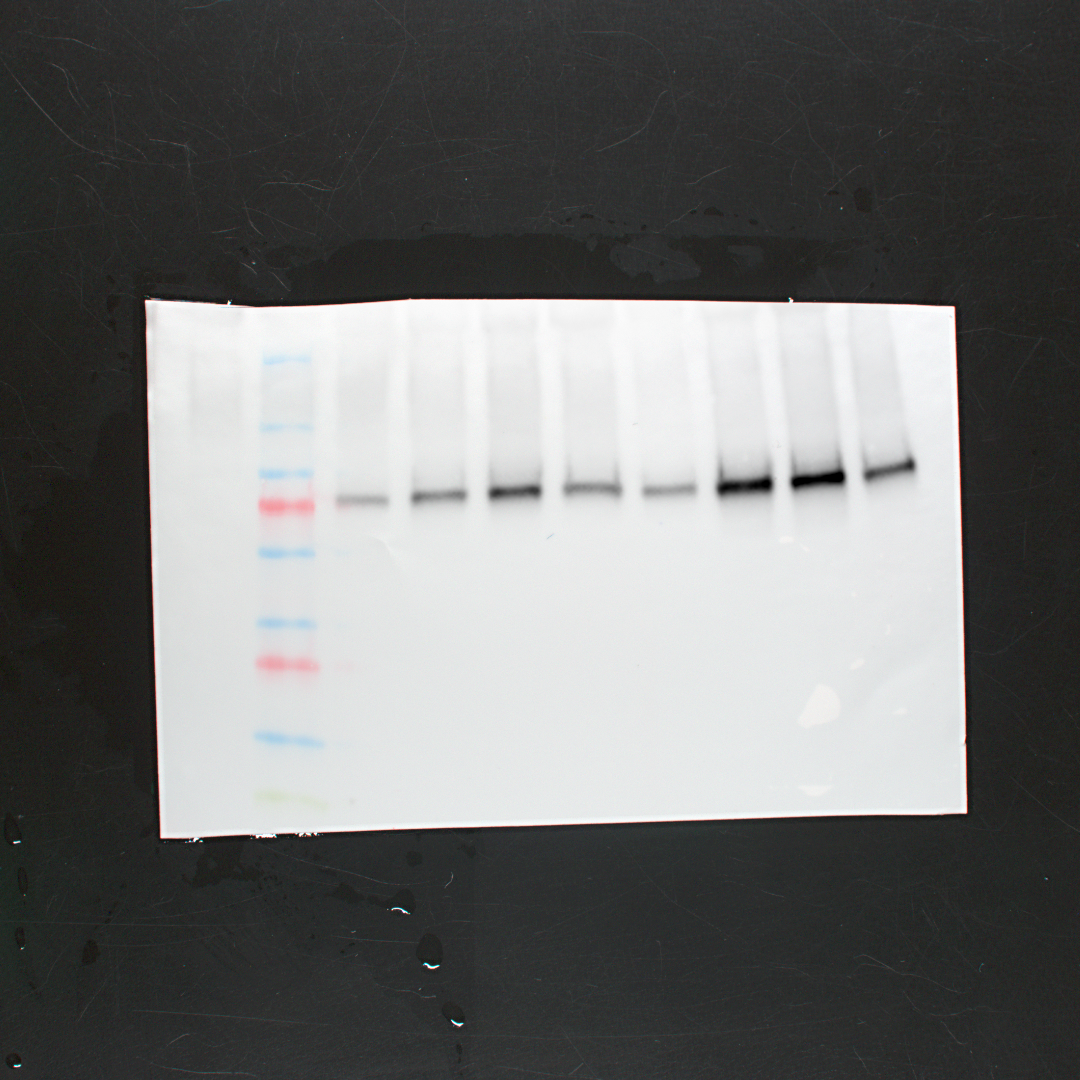

Supplement: Figure 4—figure supplement 2—source data 1. [file elife-80604-fig4-figsupp2-data1.zip › Fig 4 sup 2 /Fig 4 sup 2 A lower panel marker overlay 1.Tif]

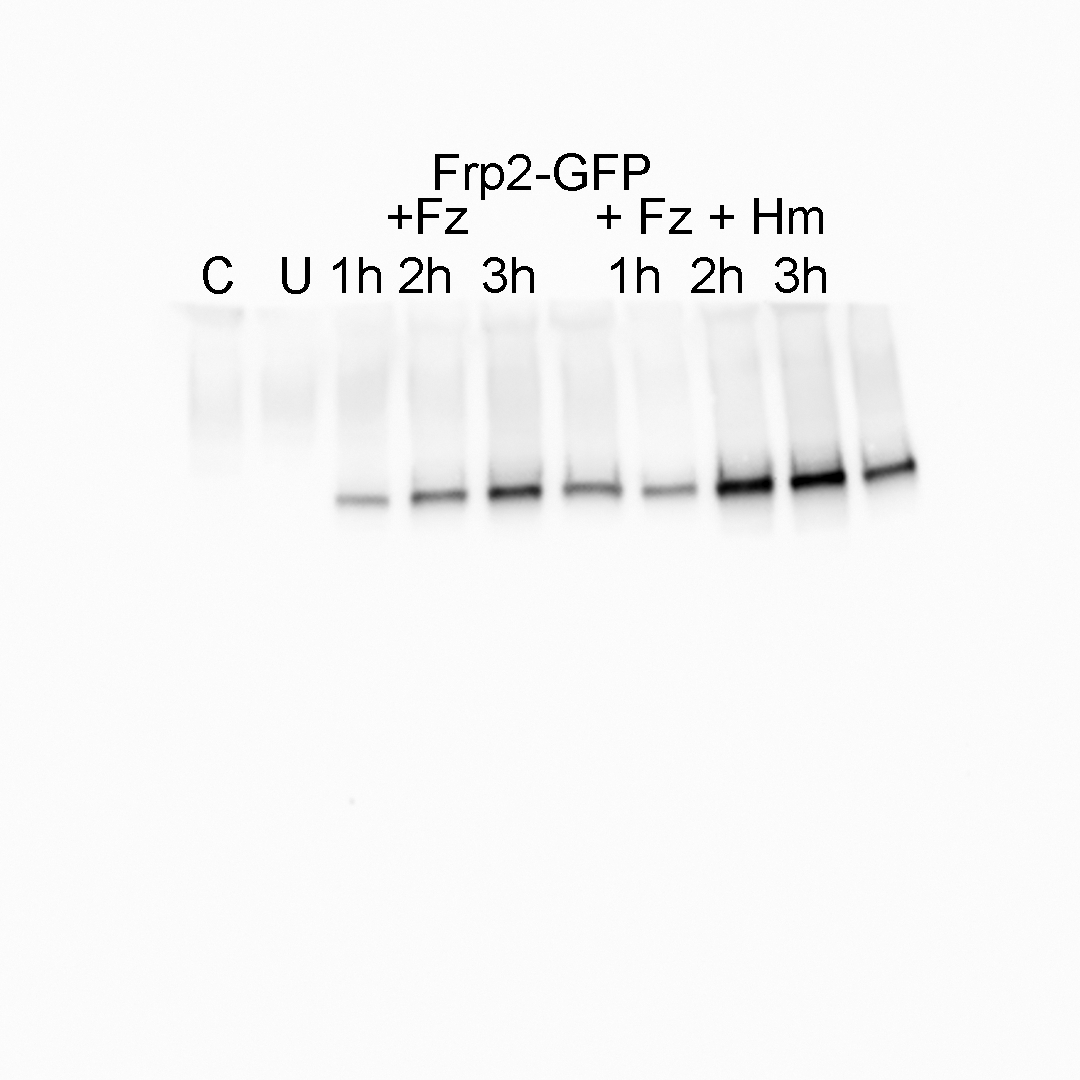

Supplement: Figure 4—figure supplement 2—source data 1. [file elife-80604-fig4-figsupp2-data1.zip › Fig 4 sup 2 /Fig 4 sup 2 A lower panel marked.Tif]

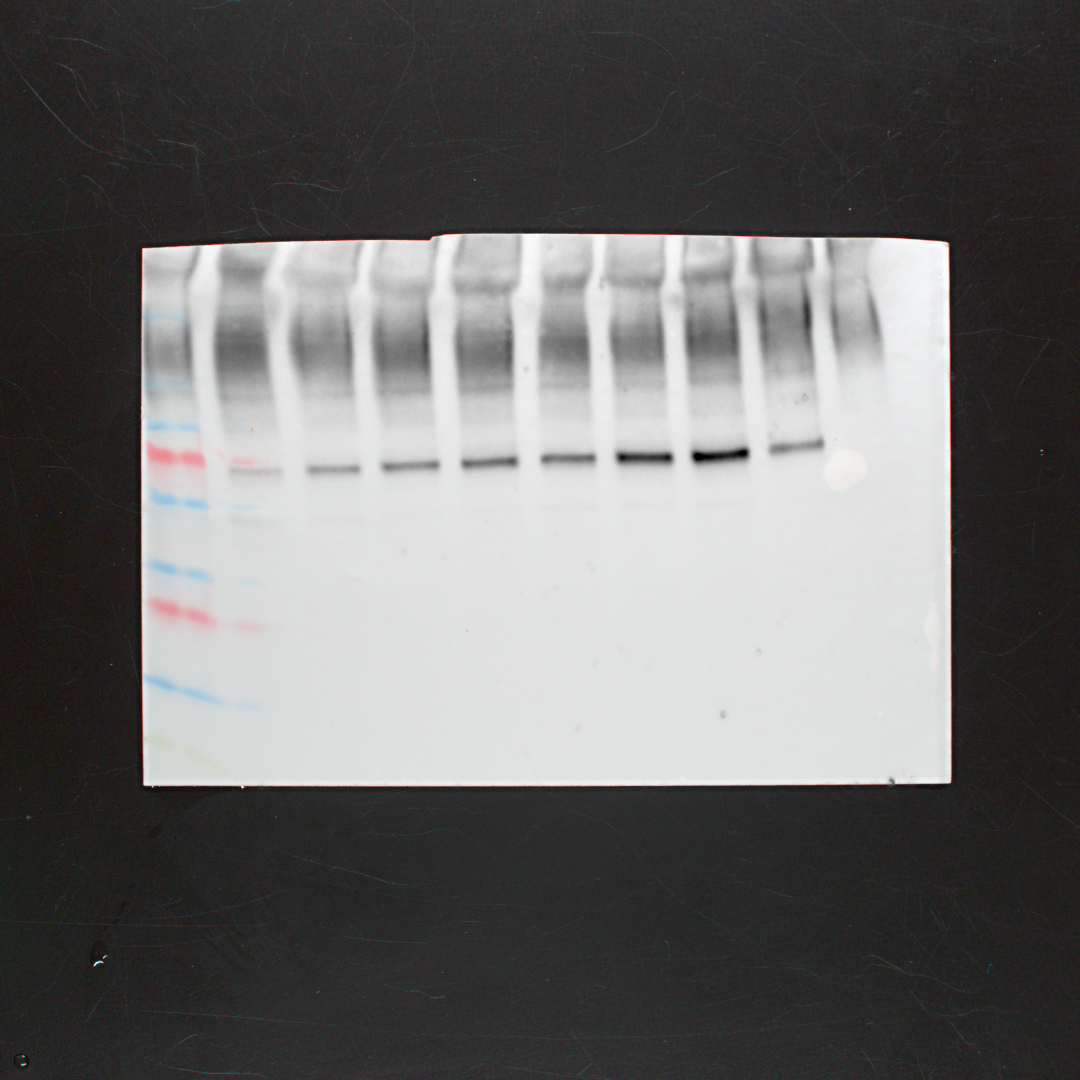

Supplement: Figure 4—figure supplement 2—source data 1. [file elife-80604-fig4-figsupp2-data1.zip › Fig 4 sup 2 /Fig 4 sup 2 A top panel marker overlay.Tif]

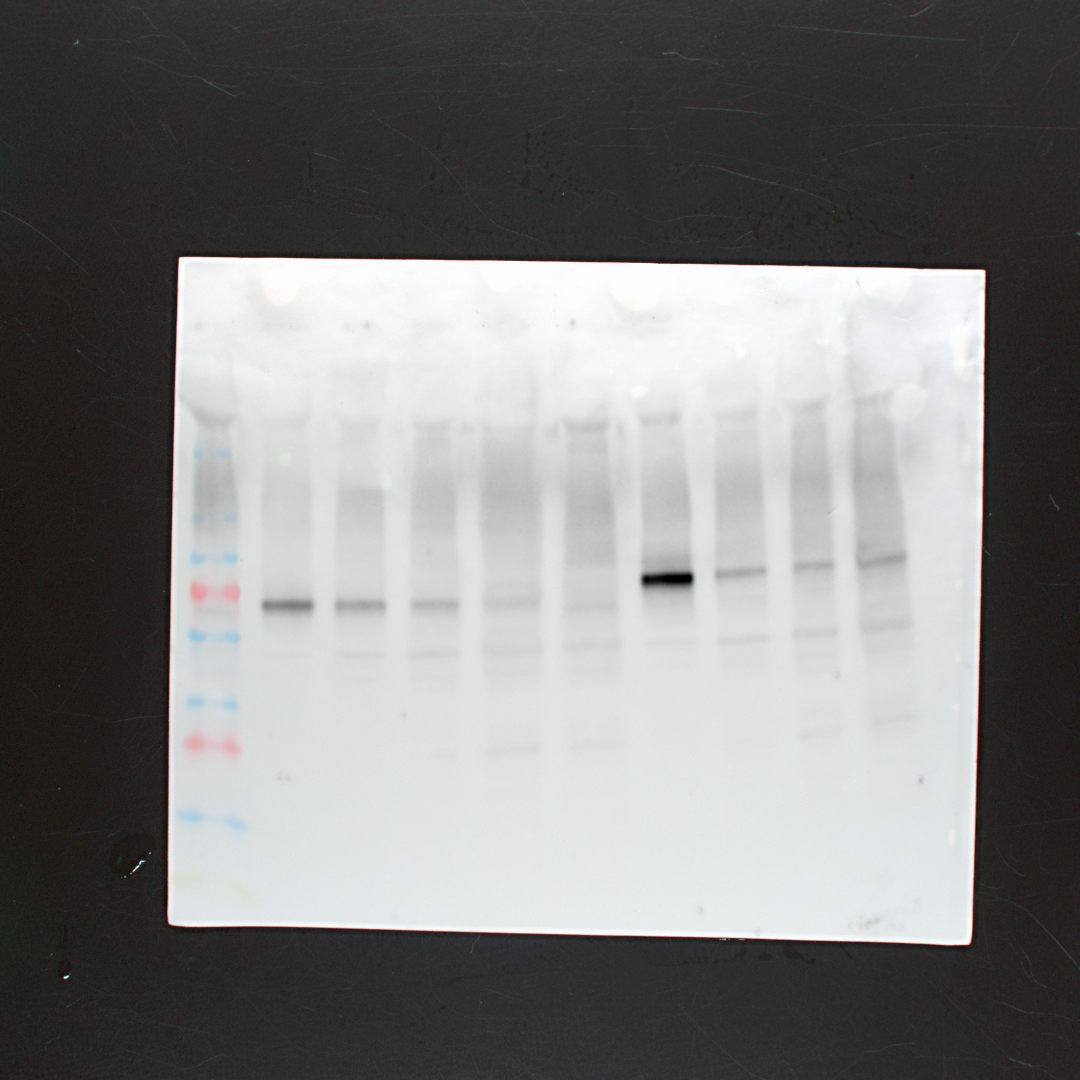

Supplement: Figure 4—figure supplement 2—source data 1. [file elife-80604-fig4-figsupp2-data1.zip › Fig 4 sup 2 /Fig 4 sup 2 B marker overlay.Tif]

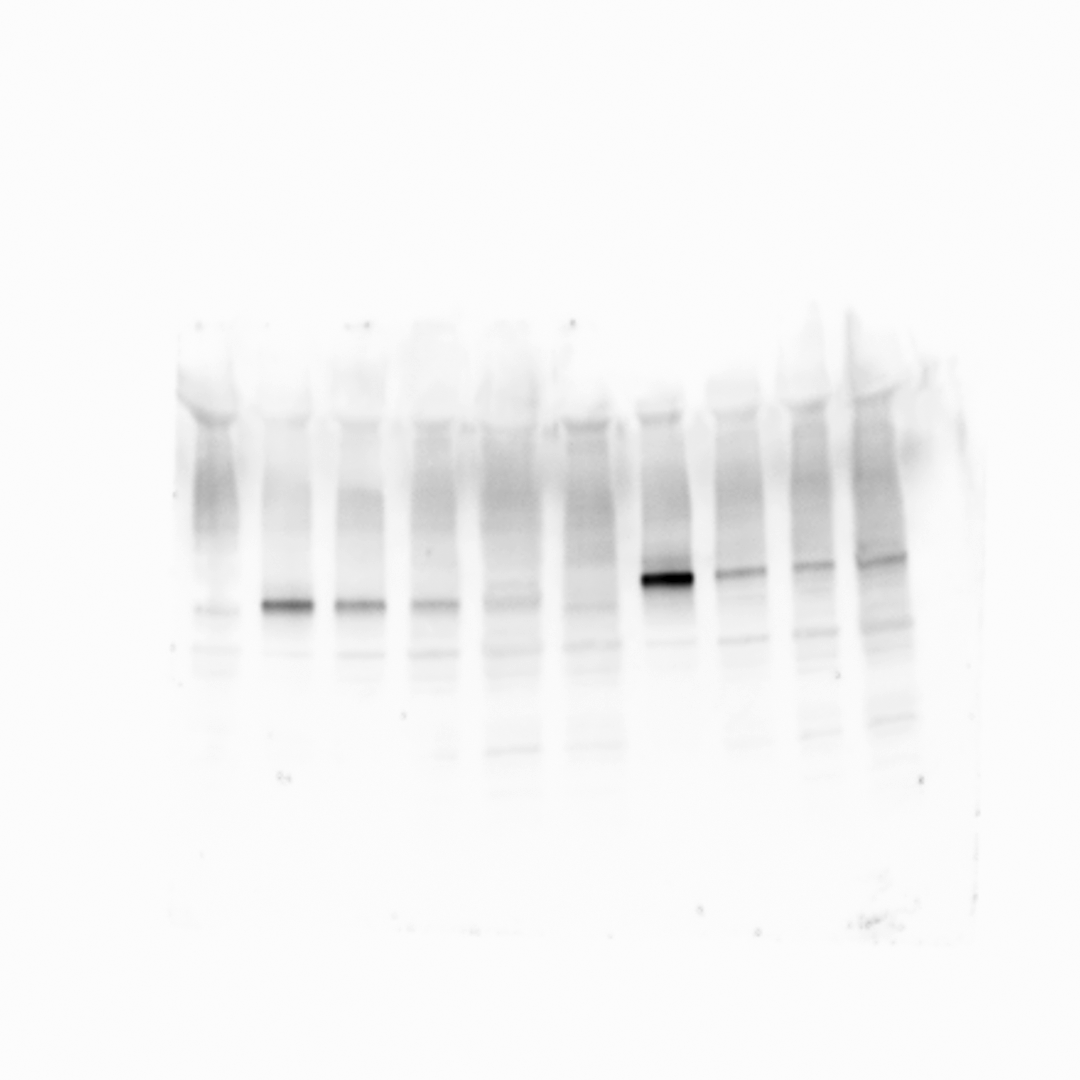

Supplement: Figure 4—figure supplement 2—source data 1. [file elife-80604-fig4-figsupp2-data1.zip › Fig 4 sup 2 /Fig 4 sup 2 B raw.Tif]

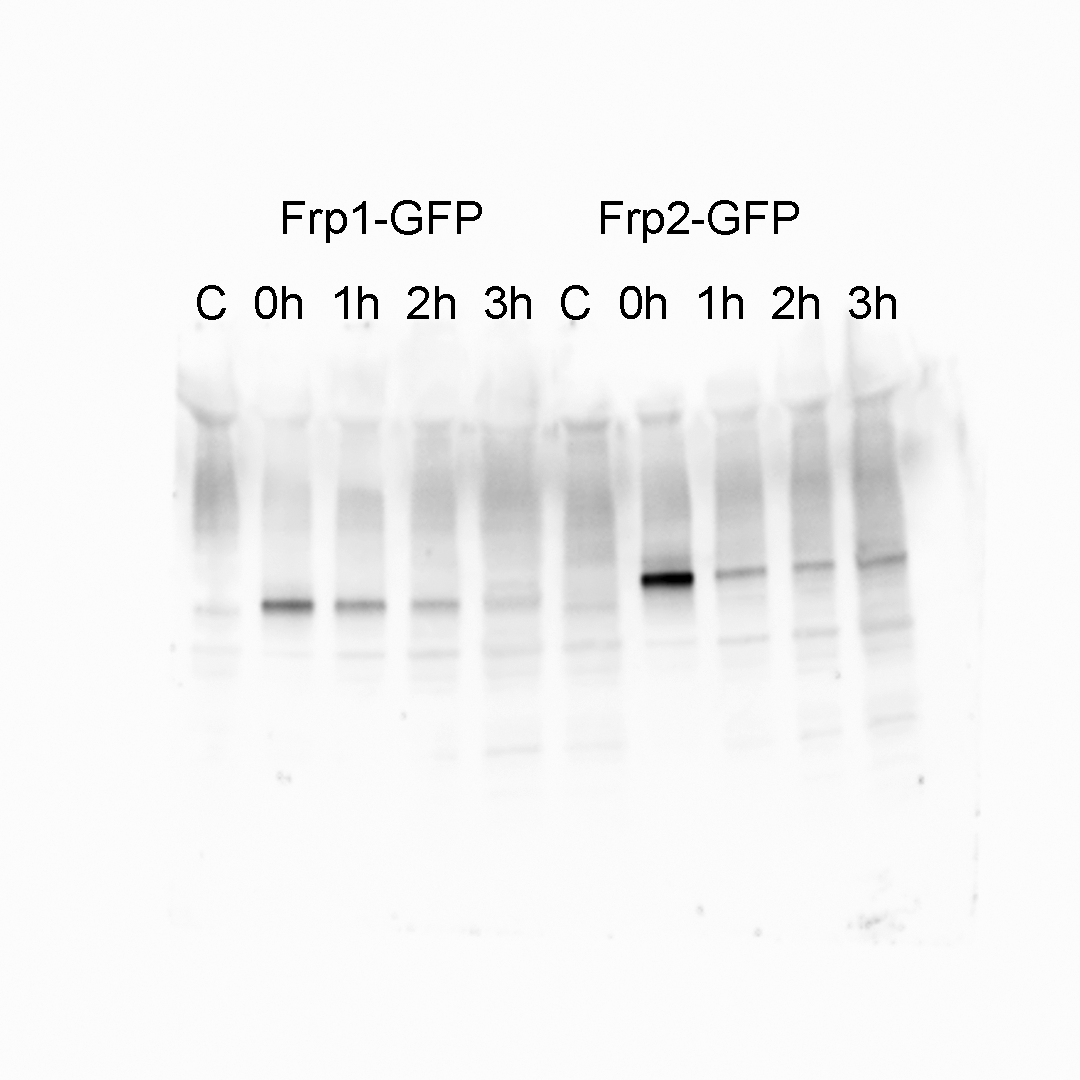

Supplement: Figure 4—figure supplement 2—source data 1. [file elife-80604-fig4-figsupp2-data1.zip › Fig 4 sup 2 /Fig 4 sup 2 B marked.tif]
