# Supplementary material for: Ferric reductase-related proteins mediate fungal heme acquisition
Source: eLife. 2022 Oct 6;11:e80604. doi: 10.7554/eLife.80604 (PMC9635878; doi:10.7554/eLife.80604)
Supplement: Supplementary file 1. [file elife-80604-supp1.docx]

Appendix 1 – Table 1

| # No: | Chain | Z | rmsd | lali | nres | %id | PDB Description |
| --- | --- | --- | --- | --- | --- | --- | --- |
| 1: | d4p2-A | 22.5 | 0.5 | 111 | 1018 | 66 | **CANAL:AF-G1UB63-F1 CELL WALL PROTEIN 1;** |
| 2: | d257-A | 22.2 | 0.2 | 111 | 147 | 100 | **CANAL:AF-Q5A0X8-F1 SECRETED HEMOPHORE CSA2;** |
| 3: | d2iu-A | 19.4 | 1.2 | 109 | 219 | 60 | **CANAL:AF-Q59UT5-F1 GPI-ANCHORED HEMOPHORE PGA7;** |
| 4: | d4k5-A | 16.8 | 0.4 | 111 | 241 | 72 | **CANAL:AF-Q59UT4-F1 GPI-ANCHORED HEMOPHORE RBT5;** |
| 5: | d49j-A | 16.2 | 0.5 | 111 | 250 | 67 | **CANAL:AF-Q59UP6-F1 GPI-ANCHORED HEMOPHORE PGA10;** |
| 6: | d4ky-A | 4.6 | 3.3 | 73 | 751 | 14 | *CANAL:AF-A0A1D8PTC0-F1 FAD-BINDING FR-TYPE DOMAIN-CONTAINING PROTEIN;* |
| 7: | d4ri-A | 4.6 | 2.7 | 67 | 271 | 19 | CANAL:AF-Q5A1M6-F1 CCW14P; |
| 8: | d23p-A | 4.5 | 2.7 | 72 | 286 | 19 | *CANAL:AF-A0A1D8PTF8-F1 FRE30P;* |
| 9: | d6ch-A | 4.4 | 3 | 75 | 739 | 16 | *CANAL:AF-Q59PZ9-F1 CFL11P;* |
| 10: | d4mm-A | 4.1 | 2.9 | 72 | 730 | 21 | *CANAL:AF-A0A1D8PTF9-F1 FAD-BINDING FR-TYPE DOMAIN-CONTAINING PROTEIN;* |
| 11: | d37u-A | 3.9 | 3.1 | 71 | 710 | 20 | *CANAL:AF-A0A1D8PN54-F1 FERRIC/CUPRIC-CHELATE REDUCTASE;* |
| 12: | d5x4-A | 3.8 | 2.8 | 65 | 449 | 5 | CANAL:AF-Q59PZ5-F1 HISTONE ACETYLTRANSFERASE GCN5; |
| 13: | d6e9-A | 3.5 | 3 | 73 | 710 | 21 | *CANAL:AF-A0A1D8PN52-F1 FERRIC-CHELATE REDUCTASE;* |
| 14: | d271-A | 3.5 | 4.5 | 68 | 1927 | 7 | CANAL:AF-A0A1D8PTE0-F1 1-PHOSPHATIDYLINOSITOL 4-KINASE; |
| 15: | d3rv-A | 3.3 | 3.3 | 78 | 168 | 3 | CANAL:AF-A0A1D8PRK2-F1 SYS1P; |
| 16: | d54e-A | 3.3 | 4 | 64 | 1847 | 5 | CANAL:AF-A0A1D8PRM4-F1 UNCHARACTERIZED PROTEIN; |
| 17: | d2hq-A | 3.3 | 3.2 | 65 | 482 | 3 | CANAL:AF-Q5AK24-F1 GPI MANNOSYLTRANSFERASE 3; |
| 18: | d5tq-A | 3.1 | 3.3 | 67 | 1665 | 10 | CANAL:AF-A0A1D8PDJ8-F1 DNA-BINDING PROTEIN; |
| 19: | d3u0-A | 3.1 | 4.4 | 74 | 1032 | 11 | CANAL:AF-A0A1D8PHC5-F1 UNCHARACTERIZED PROTEIN; |
| 20: | d2vv-A | 3 | 3.1 | 61 | 323 | 7 | CANAL:AF-Q5AD01-F1 PROTEIN-LYSINE N-METHYLTRANSFERASE; |
| 21: | d2v9-A | 3 | 4.2 | 64 | 2500 | 6 | CANAL:AF-A0A1D8PJ54-F1 UTP20P; |
| 22: | d4xm-A | 3 | 3.7 | 67 | 1195 | 9 | CANAL:AF-Q5ADW3-F1 CULLIN-ASSOCIATED NEDD8-DISSOCIATED PROTEIN 1; |
| *C. albicans* predicted protein structures most similar to the Csa2 hemophore. The CFEM hemophore descriptions are in boldface, an unrelated CFEM protein is underlined, and the ferric reductases are marked in italics. | | | | | | | |
